# Re-emergence of vaccine-derived poliovirus in Israel, US, and UK – Call for promoting the use of novel oral polio vaccine

**DOI:** 10.1016/j.amsu.2022.104778

**Published:** 2022-09-24

**Authors:** Syeda Shahnoor, Rimmel Ghaffar, Muskan Asim Taimuri

**Affiliations:** Department of Internal Medicine, Dow University of Health Sciences, Pakistan

Poliomyelitis, a disabling and potentially deadly viral disease, invades the nervous system and has paralytic manifestations [1]. Its etiological agent, Poliovirus, is a member of the genus Enterovirus, belonging to the Picornaviridae family [2]. It is a highly contagious disease and spreads from person to person mainly through contact with the faeces of an infected person, via nasal and oral sections and rarely by contaminated food or water [3]. The presentation of polio is highly variable with a broad range of symptoms. A vast majority of people who contact polio develop mild flu-like symptoms that include sore throat, fever and nausea. However, a few exhibit a more severe form of poliovirus infection that affects the brain and nerves such as weakness in muscles of the limbs causing paralysis and even respiratory failure if muscles of breathing are immobilized [1]. The 19th and 20th centuries witnessed recurrent episodes of the polio epidemic to the point it became a prominently feared disease of its time. The fear of polio erupted everywhere as this disease engulfed many regions around the globe [4]. Not only was this disease deadly but those who survived faced lifelong complications [4]. The global polio eradication initiative was established in 1988 when the wild poliovirus was endemic in 125 countries [5]. The tremendous efforts of this initiative reduced the number of polio cases by more than 99% from 350,000 people being paralyzed in 1988 to only 6 cases reported in 2021 [1]. In 1994, the WHO Region of The Americas was certified polio-free followed by the WHO Western Pacific Region in 2000. By 2014, the south-east Asia region also got the credits for eradicating polio [1]. This emphasizes the importance of immunizations which have saved billions of lives and prevented countless illnesses and disabilities across the globe.

Since there are no approved antiviral treatments for polio, vaccination is the only possible prevention. Immunity is established by the administration of two types of vaccines – Inactivated Polio Vaccine (IPV) and live attenuated Oral Polio Vaccine (OPV) [6]. OPV is more feasible to use as no professional health workers are required to administer it and is more cost-effective [7]. Hence, OPV is used in mass polio vaccination campaigns in developing countries including Pakistan. However, OPV has been known to cause Vaccine Associated Paralytic Poliovirus (VAPP) in some cases and very rarely, results in circulating vaccine-derived poliovirus (cVDPV) with a reversion of the vaccine strains to the more neurovirulent profile of wild poliovirus [7]. IPV, on the other hand, contains an inactivated form of poliovirus, hence, it does not cause infection from the virus, reducing the risk of reemergence and thus, paralysis [8]. It is used in developed countries, but it is expensive to produce and requires cold storage and intramuscular administration. Because these requirements are impractical in the developing world, an OPV is used instead [9]. However, IPV is not as effective as OPV at preventing transmission and adds too little immunity to the intestines, which can still result in infection after virus contraction [8].

While polio was eliminated from all over the world, it remained endemic in Pakistan and Afghanistan as 100% (67.5% in Pakistan, 32.5% in Afghanistan) of polio cases were reported in these two countries in the last five years. Hence it remained an active threat to the world [10]. The successful polio eradication efforts everywhere else, therefore, did not last enough since new cases in Israel in March 2022 and the US in July 2022 have been detected along with the detection of live polio virus in sewage samples in the UK in June 2022 [[11], [12], [13]]. This proves to be a forewarning for the world which is already burdened by the unprecedented covid19 pandemic and the current monkeypox outbreak and becomes a matter of interest as to why the cases of polio have re-emerged despite the use of inactivated polio vaccine (IPV). The possible explanation for the re-emergence of these cases in the US, UK, and Israel is that the vaccine-derived poliovirus (VDPV) can emerge in areas where the live oral polio vaccine is not in use [14]. The likely source of the spread of the live strains of poliovirus could have been the faeces of individuals who were vaccinated overseas and returned to their country [15]. This poses a constant threat for polio-free countries of importing a vaccine-derived poliovirus from places where OPV is used, showing that the complete eradication of polio will also involve the gradual disuse of OPV, or a severe resurgence of polio may occur otherwise.

A possible solution to this issue is the use of more genetically stable oral poliovirus vaccine strains, listed by WHO in 2020, which are less likely to regain their virulence, evolving into VDPVs [16] This is the first time an unlicensed vaccine has been granted an Emergency Use Listing status by WHO [17]. Novel oral polio vaccine type 2 (nOPV2), a genetically modified form of monovalent oral polio vaccine type 2 (mOPV2) [a type of oral polio vaccine for circulating vaccine-derived poliovirus type 2 (cVDPV2) outbreaks], has been shown to be safe and well-tolerated [18]. Because of the improved stability in key genomic segments of the vaccine virus, nOPV2 is thought to have enhanced genetic stability and is expected to have a reduced possibility of reversion to VDPV [19,20].

Considering the devastation caused by covid19 pandemic and the monkeypox outbreak, which is saturating the already burdened healthcare sectors around the globe, especially the developing countries, it is imperative to prevent a further rise in cases. To eliminate the polio cases from countries where it is endemic, nOPV2 will be an essential source for controlling the cVDPV outbreak. Polio vaccination campaigns have been particularly hampered by the lack of awareness about the lifesaving benefits of vaccines and conspiracy theories about the side effects. Hence, the lack of vaccination and incomplete vaccinations have been the main cause of the outbreak of the emergences of cVDVP. Therefore, there remains a need for mass high–quality vaccination campaigns to increase awareness about the benefits of vaccines in endemic countries and implement routine immunizations of nOPV2 which may reduce the risk of seeding new polio outbreaks. The data of vaccinated individuals should be regularly checked to ensure that vaccination campaigns meet their annual vaccination goals. More research should be conducted, and large-scale human testing should be done to prove the vaccine to be effective and safe so that it could get the approval of US and UK regulators.

## Ethical approval

Not applicable.

## Funding

None.

## Authors contribution

Syeda Shahnoor: Writing – original draft, final approval and agreeing to the accuracy of the work.

Rimmel Abdul Ghaffar: Writing – original draft, final approval and agreeing to the accuracy of the work.

Muskan Asim Taimuri: Conceptualization, final approval and agreeing to the accuracy of the work.

## Conflicts of interest

None.

## Registration of research studies

1. Name of the registry: Not applicable
2. Unique Identifying number or registration ID: Not applicable
3. Hyperlink to your specific registration (must be publicly accessible and will be checked): Not applicable

## Guarantor

Syeda Shahnoor.

Rimmel Abdul Ghaffar.

Muskan Asim Taimuri.

## Consent

Not applicable.
